# Forequarter amputation: a safe rescue procedure in a curative and palliative setting in high-grade malignoma of the shoulder girdle

**DOI:** 10.1186/s12957-016-0973-7

**Published:** 2016-08-15

**Authors:** Ulrich Elsner, Marcel Henrichs, Georg Gosheger, Ralf Dieckmann, Markus Nottrott, Jendrik Hardes, Arne Streitbürger

**Affiliations:** Department of Orthopedics and Tumororthopedics, University Hospital of Münster, Albert-Schweitzer-Campus 1, 48149 Münster, Germany

**Keywords:** Forequarter amputation, Shoulder girdle, High-grade sarcoma, Osteosarcoma, Upper extremity

## Abstract

**Background:**

Forequarter amputation (FQA) is a surgical treatment of tumors in the upper extremity and shoulder girdle that infiltrate the neurovascular bundles. In both curative and palliative settings, FQA can serve as an effective oncological treatment.

**Methods:**

This study presents the FQA-related data of 30 patients (mean age 50 years) treated between 2000 and 2012. Their medical condition was high-grade bone or soft tissue sarcoma in 26 and high-grade carcinoma in four cases.

**Results:**

Mean operation time was 119 min. One major and five minor complications occurred in the postoperative period. Resection margins were wide in 91 % of patients. Local recurrence was observed in four patients after 19 months on average. Patients treated with curative intention had a 5-year survival chance of 39 %. Average survival after palliative amputation was 11 months.

**Conclusions:**

FQA provides an opportunity for adequate oncological margins in large tumors, while offering relief from tumor-induced distress in palliative situations.

## Background

In most cases of primary and secondary musculoskeletal tumors, a limb salvage procedure for local treatment is available. Therefore, few tumors require an amputation [1]. While amputations of the lower extremity can be sufficiently compensated by exoprostheses [2, 3], the psychological and physiological impact of a forequarter amputation (FQA) on the patient is significant. However, only a small number of tumors around the shoulder girdle infiltrating the nerve and vessel bundle require this type of amputation to achieve sufficient oncological margins in a curative or even in a palliative setting. Although FQA is an extensive operation, oncologically satisfying results are achievable [4, 5]. The use of exoprostheses to regain the functions of the lost arm, however, is not very common among patients. Typically, only a shoulder gab is applied to cosmetically restore the shoulder contour.

The goal of the present study was to develop a clinical guideline, what type of patients benefit from FQA. To that end, we compiled data from 30 patients who underwent an interscapulothoracic resection in our institution during the past 12 years. We analyzed these FQAs in regard to perioperative risks and oncological outcome.

## Methods

**Fig. 1:**
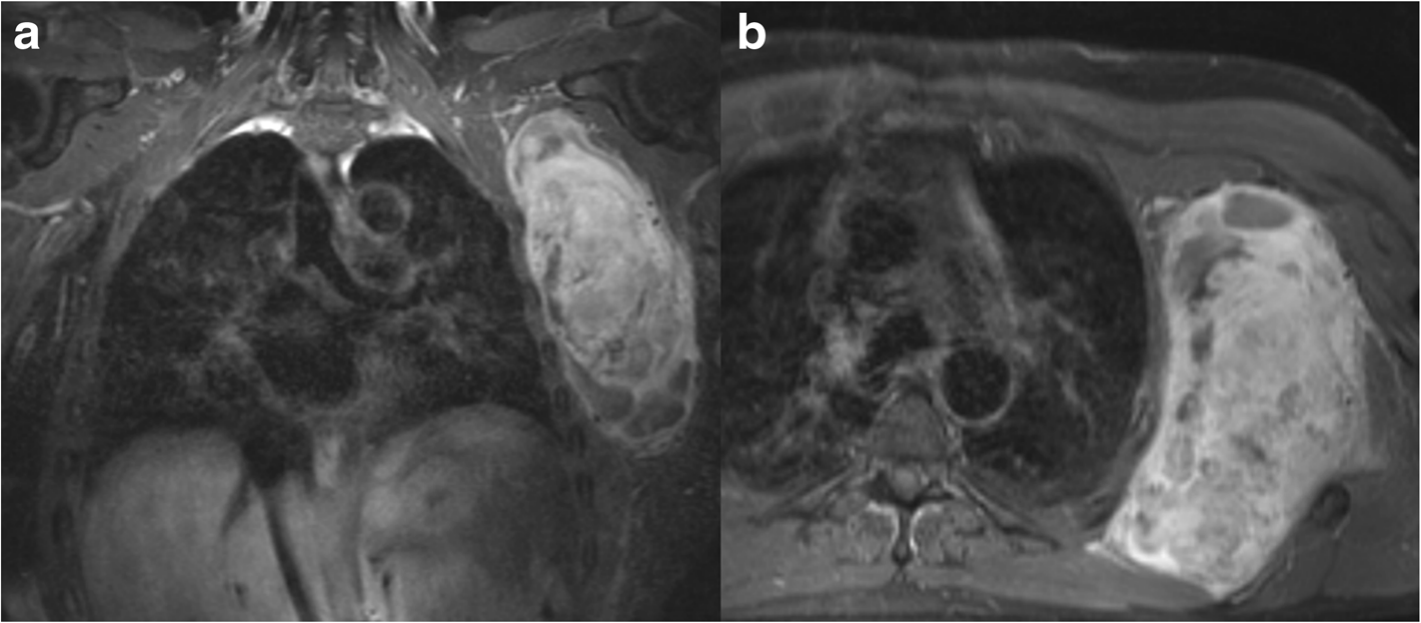
**a**, **b** MRI T1 with gadolinium enhancement (3a coronal plane/3b axial plane) imaging of a 77-year-old female with a radiotherapy-induced osteosarcoma of the glenoid (37 years after breast cancer treatment). The MRI shows the infiltration of the thoracic wall and a tumor encasement of the brachial vessel/nerve bundle

Between 2000 and 2012, we performed FQA in 30 patients (11 females, 19 males) suffering from high-grade malignancies of the shoulder girdle (Table 1). Their median age at FQA was 50 years (range 8–83 years). FQA was performed in case of locally recurrent tumor in 17 patients and for primary tumor manifestation in 13 patients. Tumor stage according to the Enneking staging system [6] was stage IIB in 14 and stage III in 12 patients. Three patients showed an advanced metastatic stage of carcinoma, and one patient suffered from a large single bone metastasis of a bronchial carcinoma. Fifteen patients had a primary soft tissue tumor, whereas 15 patients had a primary bone involvement of the proximal humerus and/or the scapula. Pathological fracture of the humerus was present in seven patients at the time of initial diagnosis. Six of them were osteosarcoma and one chondrosarcoma (Table 1). Thoracic-wall infiltration was present in two patients (Fig. 1a, b). In both cases, a partial thoracic-wall resection was performed in addition to the FQA, and defect closure was achieved by a devitalized porcine skin graft. Resection margins were categorized as wide, marginal, or intralesional, following the Enneking classification based on the histopathological examination of the specimen [7]. The mean residence time in hospital was 19.5 days (range 9–59 days). In all cases but one, postoperative observation on intermediate or intensive care unit was necessary. All but one patient with bone and soft tissue sarcoma received chemotherapy. Overall, 18 patients underwent additional local radiotherapy; 28 patients had a median follow-up of 42 months (range 1–195 months) after FQA (Table 1). Median follow-up after initial diagnosis was 68 months (range 3–292 months). Two patients were unavailable to follow up after their discharge from the hospital. Both patients returned abroad to their home countries (2 months after FQA).

**Table 1:** Patient-related synoptical table

Patient	Gender	Age at FQA	Diagnosis	Prior surgery	Tumor origin	Staging at time of FQA	Pathological fracture	Indication	Treatment intention	Metastases before FQA	Local recurrence (months after FQA)	Follow-up (months)	Status
1	m	32	es OS	y	st	II B	n	iT	c	n	58	101	AWD
2	f	77	OS	n	b	II B	n	iT	c	n	n	3	DOD
3	f	72	OS	y	b	II B	n	TR	c	n	n	136	NED
4	f	19	OS	y	b	III B	n	TR	c	y (lung)	n	72	DOD
5	m	16	OS	n	b	II B	y	iT	c	n	n	132	NED
6	m	19	OS	n	b	III B	y	iT	c	y (lung)	n	2	LOF
7	m	55	Lung cancer	n	b	Single bone metastasis	n	iT	c	y	n	195	NED
8	m	19	OS	y	b	III B	n	TR	c	y (lung)	n	13	DOD
9	f	63	DD CS	y	b	III B	y	iT	c	y (lymph node)	2	60	DOD
10	f	64	MFG G III	y	b	II B	n	TR	c	n	n	5	DOD
11	m	32	OS	n	b	II B	y	iT	c	n	n	17	DOD
12	f	10	OS	y	b	III B	y	iT	c	y (lung)	n	109	NED
13	f	69	NOS G III	n	st	II B	n	iT	c	n	n	58	NED
14	m	57	Liposarcoma G III	n	st	II B	n	iT	c	n	n	38	DOD
15	m	32	Hemagioendothelioma	y	b	II B	n	TR	c	n	n	91	NED
16	m	43	MFH G III	y	st	II B	n	TR	c	n	n	8	DOD
17	f	35	CS G III	n	b	II B	n	iT	c	n	n	2	LOF
18	f	76	MFH G III	y	st	II B	n	TR	c	n	11	26	DOD
19	m	74	Liposarcoma G III	y	st	II B	n	TR	c	n	n	31	DOD
20	m	73	CS G II	y	st	III B	n	TR	c	y (lymph node)	n	25	DOD
21	m	66	NOS G III	y	st	III B	n	TR	c	y (lung)	n	41	NED
22	f	16	OS	n	b	III B	y	iT	c	y (lung, bone)	n	10	DOD
23	m	8	OS	n	b	III B	y	iT	c	y (bone)	n	9	DOD
24	m	37	Fibrosarcoma G III	y	st	II B	n	TR	c	n	4	18	DOD
25	m	76	MFH G III	y	st	III B	n	TR	p	y (lung)	n	19	DOD
26	m	74	MFH G III	y	st	III B	n	TR	p	y (lung)	n	15	DOD
27	m	83	Squamous cell carcinoma	y	st	Multiple metastases	n	TR	p	y (lymph node, lung)	n	1	DOD
28	m	53	Synovial sarcoma G III	y	st	III B	n	TR	p	y (lung)	n	21	DOD
29	f	70	Merkel cell carcinoma	y	st	Multiple metastases	n	TR	p	y (lymph node)	n	10	DOD
30	m	82	Squamous cell carcinoma	y	st	Multiple metastases	n	TR	p	y (lymph node)	n	17	NED

*OS* osteosarcoma, *es OS* extraskeletal osteosarcoma, *DD CS* dedifferentiated chondrosarcoma, *st* soft tissue, *b* bone, *iT* initial surgery, *TR* tumor recurrence, *c* curative, *p* palliative, *DOD* dead of disease, *AWD* alive with disease, *LOF* lost to follow-up, *NED *no evidence of disease

The Short Form (36) Questionnaire (SF-36) questionnaire was used to evaluate the postoperative functional and psychological results after FQA; it was also available for the nine surviving patients.

The postoperative pain level was measured on a six-step scale according to the SF-36 questionnaire (1-none, 2-very, 3-mild, 4-moderate, 5-severe, 6-very severe) and was available for 27 of the patients.

## Results

The average duration of surgery was 119 min (range 36–240 min). Patients with primary tumor manifestation had a shorter operation time than patients with recurrent tumor (108 vs. 126 min). The mean amount of blood transfusion needed was 1.1 packed blood cells (PBC) (range 1–6). There was no difference observed between primary and recurrent tumors. Primary wound closure was achieved in all patients.

Regarding the overall surgical risk of the FQA, we observed one major and five minor complications in the perioperative setting. Also, there was no intraoperative complication noticed. The major complication was seen in a patient who developed a pneumothorax with consecutive respiratory insufficiency after additional partial thoracic-wall and lung resection. The patient needed intensive care unit (ICU) treatment for 2 weeks. However, this 77-year-old patient did not recover and died 3 months after surgery due to generally weak condition. The minor complications were delayed wound healing and the need of surgical local debridement in all five patients. However, wound closure was achieved for all patients at the latest follow-up. Four of the five wound complications occurred after initial local radiation therapy.

Initial postoperative treatment of pain was realized by continuous interscalene brachial plexus block with Bupivacaine 0.175 % in all patients [8]. Postoperative phanthom pain and/or local pain were observed in all patients. An average pain intensity of 3.07 on the six step scale was perceived. Although most of the patients required the daily use of per oral analgetic drugs, only 28 % (8/28) suffered of moderate (5/28) or even more intensive pain (3/28).

The evaluation of the SF-36 questionnaire presented fair results in terms of psychological and functional outcome. The mean mental condition parameter (MCS) was 47.7 (range 25.5–61.2), and the functional parameter (PCS) was less favorable with a mean PCS of 37.7 (range 13.7–54.8) as expected.

**Fig. 2:**
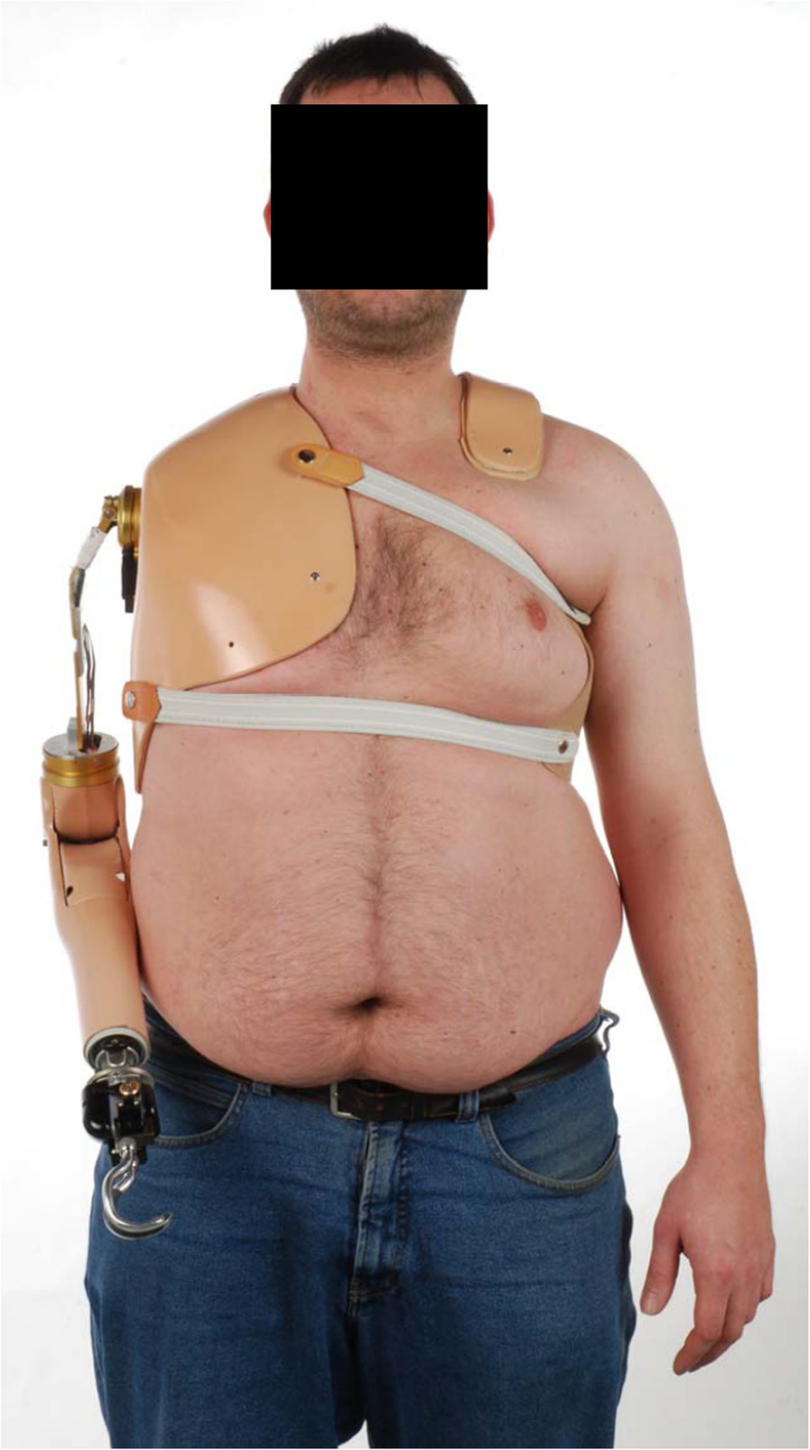
35-year-old patient using a myoelectric exoprosthesis after FQA because of a recurrent hemangioendothelioma

Only two of the nine surviving patients are using a shoulder gap on a regular basis, and one patient uses a myoelectric arm exoprosthesis with a satisfying function (Fig. 2). The other six patients did not use any device. Three of the other 19 patients used a shoulder gap regularly, and 3 others were using a cosmetic arm replacement only.

**Fig. 3:**
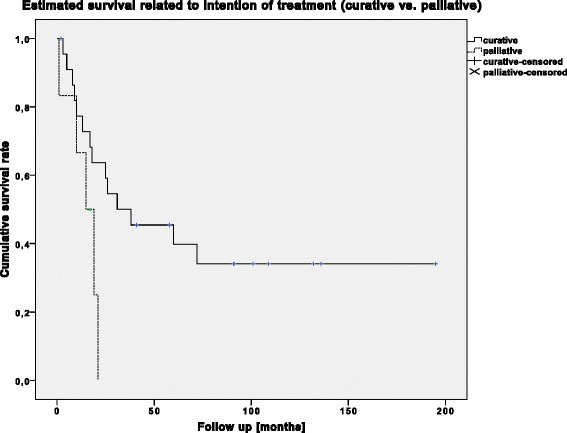
Cumulative survival related to treatment intention

Regarding the oncological outcome, the patients are divided into two treatment groups, depending on the underlying treatment intention. Before FQA, a curative treatment strategy was set in 24 patients (80 %) and 6 patients (20 %) were classed to be palliative. Overall survival differed significantly (*p* = 0.041) between these two groups (Fig. 3).

### Curative group

Single metastasis at the time of FQA was observed in 9 (37.5 %) of the 24 patients with a curative treatment goal. All of them underwent resection of the pulmonary and lymph node metastasis, so that the intent of treatment was defined curative; 88 % received additional chemotherapy and 50 % local irradiation. Twelve of these 24 patients had newly diagnosed tumors, and 7 of them had a pathological fracture of the proximal humerus at the time of initial diagnosis. In the other 12 patients, FQA was performed because of recurrent tumor. In the curative treatment group, wide resection margins were achieved in most patients (22/24) and marginal resection margins in two patients (9 %). Two patients were unavailable for potential follow-up.

Local recurrence of the tumor occurred in four patients (16 %) after an average 19 months (range 1–58 months) post FQA. All four were treated with the FQA because of recurrent tumor after previous local tumor excision. None of the patients that were treated primarily with the FQA experienced a local relapse; 14 patients (58 %) died of disease after an average 24 months (range 3–72 months) following FQA. One patient is still alive with disease (pulmonary metastasis) 101 months after treatment. The other seven patients are without evidence of disease at the latest follow-up (on average, 103 months after FQA and 134 months after initial diagnosis).

**Fig. 4:**
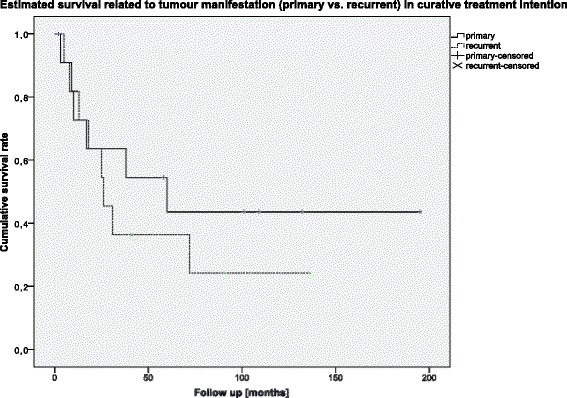
Cumulative survival in the curative treatment group related to primary vs. recurrent tumor presentation

Regarding the oncological outcome in terms of local recurrence or survival, there was no statistically significant difference observed between patients affected by initial tumor versus recurrent tumor. Five-year survival was 39 % for all patients subject to curative treatment. For patients with primary tumor manifestation, the 5-year survival was 43.6 % and for recurrent tumor 34.1 % (*p* = 0.204) (Fig. 4).

### Palliative group

All six patients classified as palliative had a recurrent tumor and multiple non-resectable metastases at the time of FQA. In this subgroup, one to four local tumor surgeries were performed prior to FQA. The mean time from initial surgical treatment to final FQA was 37 months (range 2–112 months). Local tumor exulceration (Fig. 1a, b) and untreatable pain as well as malfunction or dysfunction of the arm due to tumor infiltration were motivations to perform FQA in all of these patients. In this group, resection margins were wide in three, marginal in one, and intralesional in two cases. All six patients received local irradiation after FQA, although four patients had already received local radiation treatment after their initial surgery. Interestingly, none of them developed another local recurrence until the follow-up. However, after an average of 11 months (range 1–21 months), 83 % (5/6) of treated patients died of disease.

## Discussion

As the patient population underlying this study was small, highly selective, and heterogeneous in terms of the entity (primary bone and soft tissue tumors, carcinoma metastasis), tumor stage, and clinical presentation, the results are difficult to compare with published data. Therefore, we present key findings of our series in consideration of the surgical procedure and the oncological usefulness to develop a clinical guideline for selecting patients that may benefit from FQA. FQA needs to be considered as a treatment option in oncological surgery in highly selected cases and in a variety of high-grade soft tissue and bone sarcomas, and even in metastasis of carcinoma. For patients under curative or palliative treatment intention, FQA provides the opportunity of achieving wide oncological resection margins, also in difficult tumor presentations as described in this study and in the literature [5, 9–11].

### Surgery-related complications

An average surgery time of 119 min and the requirement of less than an average of two PBC (range 0–6) prove the low surgical risk even for critically ill patients. These findings are comparable to the results presented in literature [9, 12, 13]. Further, the low occurrence of major (3 %) and minor complications (13 %) confirms the reliability of this procedure. Similar frequencies of complications are described in a study by Baghia et al. [12], who report 20 % procedure-related complications, including three wound healing complications and one pleural effusion.

### Function

As FQA has a massive impact on the psychological and functional integrity of the patient, the potential risks must be carefully weighed against the values of this operation for each patient. We saw a curative treatment intention in 80 % of the patients included in this study, even if they had a solitary metastasis at the time of FQA, or for patients with recurrent tumor manifestation of high grade sarcoma. Despite the reduced prognosis of these particular patients, we agreed to maximum surgical and oncological treatment, in order to seize the opportunity of cure, even if it was expected to be small. In this curative concept, wide resection margins, resection of all tumor manifestation, and additional chemo- and/or radiotherapy are indispensable. In 91 % of the curative group, wide resection margins according to Enneking were achieved. Further, a 5-year survival chance of 43 % for patients with primary tumors and 34 % for patients with recurrent tumors underlines the benefits of this treatment concept for selected patients—something which has also been shown in previous studies [9, 12]. For instance, Baghia et al. [12] report 30 % survival for curatively treated patients in which a curative treatment option was only set for patients without metastasis at the time of FQA. Qadir et al. [11] reasoned: “In a select group of patients, FQA remains a relatively safe a reliable procedure for curative or palliative treatment of upper extremity malignant disease, when other less radical options has failed.” The group of six patients with a pathological fracture of a primary osteosarcoma presented an ambivalent oncological outcome. Although the pathological fracture is a clearly negative prognostic factor for survival and local recurrence [14, 15], we observed no local recurrence. Four patients died of metastatic disease. Two patients were alive without evidence of disease after a mean follow-up of 59 months after FQA. These results seem to support the decision of performing a FQA in these particular patients to achieve local tumor control and maintain the chance of cure under difficult conditions.

Despite adequate oncological margins in more than 90 % of patients, we observed a local recurrence of 16 % (4/24) in the curative treatment group, which is disappointingly high. This can be explained by the fact that all four patients suffering of a local relapse had had previous tumor surgery. In these cases, a wide oncological margin is more difficult to achieve due to the potential tumor cell contamination of the surrounding tissue and possible undetected lymph node metastasis caused by previous surgical treatment. However, it should be acknowledged that none of the patients treated initially with a FQA experienced a local relapse. Other authors have reported local recurrence frequencies of 18 % and up to 35 % after FQA [5, 9], demonstrating the difficulty of local tumor control in these particular patients.

### Palliative group

In contrast to a curative treatment intention, in which maximum treatment is imperative, the selection of patients with a palliative tumor condition has to be reconsidered even more critically. FQA may reduce tumor-related complications like pain, functional impairment, or tumor exulceration, but will not improve survival. In this study, all but one of the palliatively treated patients died within 11 months after the operation. Based on these results from a palliative setting, FQA should only be considered, if all other treatment options, such as radiotherapy, have been attempted [11].

FQA should be used to reduce the tumor-related distress and improve quality of life, which was achieved for all palliative patients in our series. In fact, this precondition is widely accepted; for example, Wittig et al. [13] presented eight patients in their study, which received a FQA with palliative intention. They all had severe, pronounced pain and a useless extremity due to the tumor expansion before surgery. All these patients experienced significant pain relief and improvement in quality of life after FQA. Although the average patient’s survival was 5.5 months (range 3–12 months) after surgery, they concluded that the FQA would be an adequate therapy [13]. In contrast to this result, Daigeler et al. [9] performed a study with patients who had preexisting pain before proximal major amputations. They reported persisting pain after operative treatment at different intensity for all patients except one [9]. Pain relief via amputation was achieved in 46 % of the treated patients only. A notable fraction of 41 % would not agree to undergo this surgical procedure again. This problem is in line with our own study results. We also observed persistent postoperative pain at varying intensity. However, all palliatively treated patients were relieved of the local tumor-related distress after the FQA. The satisfying SF-36 results support this conclusion. Obviously, the physical status classification (PSC), representing the physical status after FQA, was limited compared to other types of amputation. The mental status represented by the average mental status classification (MSC) value was comparable to patients suffering the loss of the lower extremity [16, 17].

## Conclusions

FQA provides not only a safe and reliable treatment option for patients with high-grade bone and soft tissue malignoma in primary but also recurrent tumor manifestation. The perioperative risks are low, and adequate oncological margins are achieved regularly. However, with respect to the strong impact, the indication for a FQA has to be evaluated on an individual basis. Despite the fact that palliative amputations reduce tumor-related distress, it should remain a last-resort treatment.

## Abbreviations

FQA, forequarter amputation; ICU, intensive care unit; MSC, mental status classification; PBC, packed blood cells; PSC, physical status classification; SF-36, Short Form (36) Questionnaire
